# Direct Quantitation of SARS‐CoV‐2 Virus in Urban Ambient Air via a Continuous‐Flow Electrochemical Bioassay

**DOI:** 10.1002/advs.202301222

**Published:** 2023-05-24

**Authors:** Fuze Jiang, Bei Liu, Yang Yue, Yile Tao, Zhen Xiao, Meng Li, Zheng Ji, Jiukai Tang, Guangyu Qiu, Martin Spillmann, Junji Cao, Lianjun Zhang, Jing Wang

**Affiliations:** ^1^ Institute of Environmental Engineering ETH Zürich Zürich CH‐8093 Switzerland; ^2^ Advanced Analytical Technologies Empa Dübendorf CH‐8600 Switzerland; ^3^ Guangzhou Laboratory Guangzhou International Bio Island Guangzhou Guangdong 510005 China; ^4^ School of Environment Harbin Institute of Technology Harbin 150090 China; ^5^ Institute of Systems Medicine Chinese Academy of Medical Sciences & Peking Union Medical College Beijing 100005 China; ^6^ Suzhou Institute of Systems Medicine Suzhou 215123 China; ^7^ Zurich Instruments AG Zürich CH‐8005 Switzerland; ^8^ School of Geography and Tourism Shaanxi Normal University Xi'an 710119 China; ^9^ International Joint Research Centre of Shaanxi Province for Pollutant Exposure and Eco‐Environmental Health Xi'an 710062 China; ^10^ Institute of Atmospheric Physics Chinese Academy of Sciences Beijing 100029 China

**Keywords:** bioassay, electrochemical biocircuit, SARS‐CoV‐2 bioaerosols, signal amplification, urban ambient air

## Abstract

Airborne SARS‐CoV‐2 virus surveillance faces challenges in complicated biomarker enrichment, interferences from various non‐specific matters and extremely low viral load in the urban ambient air, leading to difficulties in detecting SARS‐CoV‐2 bioaerosols. This work reports a highly specific bioanalysis platform, with an exceptionally low limit‐of‐detection (≤1 copy m^−3^) and good analytical accordance with RT‐qPCR, relying on surface‐mediated electrochemical signaling and enzyme‐assisted signal amplification, enabling gene and signal amplification for accurate identification and quantitation of low doses human coronavirus 229E (HCoV‐229E) and SARS‐CoV‐2 viruses in urban ambient air. This work provides a laboratory test using cultivated coronavirus to simulate the airborne spread of SARS‐CoV‐2, and validate that the platform could reliably detect airborne coronavirus and reveal the transmission characteristics. This bioassay conducts the quantitation of real‐world HCoV‐229E and SARS‐CoV‐2 in airborne particulate matters collected from road‐side and residential areas in Bern and Zurich (Switzerland) and Wuhan (China), with resultant concentrations verified by RT‐qPCR.

## Graphical abstract

The rollback of COVID‐19 restriction measures in public space increases potential infection risks for airborne transmission of SARS‐CoV‐2, particularly for enclosed or poorly ventilated spaces. Continuous surveillance of airborne coronavirus is critically required. This is the first study to develop novel electrochemical bioanalytical platform for direct and accurate quantitation of real‐world airborne SARS‐CoV‐2 virus in the air.

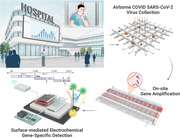

## 1 Introduction

The spread of COVID‐19 is still threatening global public health. Even in countries where high vaccination rates are achieved, breakthrough infections have been frequently reported.^[^ ^1^ ^]^ Accumulative evidence suggests that aerosol transmission is critical for spreading the SARS‐CoV‐2 virus. Airborne SARS‐CoV‐2 particles with an aerodynamic diameter less than 10 µm can remain suspended, stay infectious in the air for a prolonged time, and travel over significant distances.^[^ ^2^ ^]^ Continuous and on‐site surveillance of airborne SARS‐CoV‐2 virus in public spaces is essential for conducting a real‐time infection risk assessment of aerosol transmission and providing early warning to citizens.^[^ ^3^ ^]^ Airborne SARS‐CoV‐2 virus quantitation faces challenges in complicated aerosol sampling and low viral load in the urban ambient air.^[^ ^4^ ^]^

Various technologies have been exploited for detecting airborne SARS‐CoV‐2 virus: immunological assay‐based receptor‐binding domain (RBD) antigen detection and molecular‐assay based nucleic acid analysis.^[^ ^2^, ^4^, ^5^ ^]^ Immunological assays, such as enzyme‐linked immunosorbent assays (ELISAs) and lateral flow assay (LFA), have been reported to be integrated into air‐sampling systems for qualitative analysis of environmental COVID‐19 RBD antigens (https://www.acebiolab.com, https://www.prognosis‐biotech.com). Meanwhile, the assays demonstrate limited analytical performance when detecting low antigen load,^[^ ^4b^ ^]^ leading to difficulties to detect a trace amount of SARS‐CoV‐2 virus in the urban ambient air. Continuous mutation of SARS‐CoV‐2 also requires the assay to change its sensing biomarker frequently. Molecular‐assay‐based technologies, such as reverse transcription‐quantitative polymerase chain reaction (RT‐qPCR) and droplet digital polymerase chain reaction (ddPCR), have been used for detecting the SARS‐CoV‐2 virus from patient samples and environmental specimens.^[^ ^6^ ^]^ Techniques relying on PCR‐assisted fluorescence detection are time‐consuming and require sophisticated and expensive equipment, and trained technicians, are thus limited to laboratory‐based testing. A portable, easy‐to‐handle, robust technique is in demand for quantifying SARS‐CoV‐2 bioaerosols, allowing on‐site detection.

In this regard, electrochemical (EC) sensors benefit from their competitive advantages of being portable, cost‐effective, highly sensitive, and facile‐to‐fabricate, which are widely used for detecting biological analytes.^[^ ^7^ ^]^ Protein‐ and DNA‐based EC bioelectronics have proven their merits as bioanalytical devices to detect the biomarkers specific to COVID‐19, including SARS‐CoV‐2 antigens (S and N proteins) and their induced antibodies (S1‐lgG and S1‐lgM), and genes of N, S, ORF 1ab region.^[^ ^8^ ^]^

Here we exploited a deployable continuous‐flow multichannel EC bioassay to detect and quantify multiple viruses in urban ambient air, such as human coronavirus 229E (HCoV‐229E) and SARS‐CoV‐2. Our system demonstrated analytical accordance with RT‐qPCR and an exceptional limit of detection (LoD, 0.1 copy µL^−1^) that could discern less than 1 copy of SARS‐CoV‐2 virus in 1 m^3^ of collected air. We cultivated and aerosolized the HCoV‐229E virus to simulate the airborne transmission of COVID‐19 and used it to evaluate our system and elucidate the transmission characteristics. The system successfully quantitated HCoV‐229E and SARS‐CoV‐2 in airborne particulate matter (PM) samples, in favorable agreement with RT‐qPCR.

## 2 Results and Discussion

### 2.1 RNA Isolation from SARS‐CoV‐2 Bioaerosols

**Figure 1:**
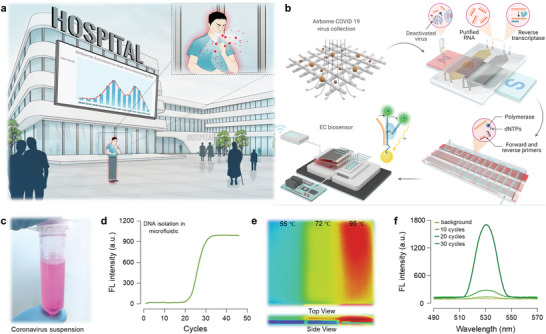
Continuous‐flow EC bioassay for the quantitation of airborne SARS‐CoV‐2 virus. a) Illustration of airborne SARS‐CoV‐2 virus transmission from infected individuals in a public space and speculative airborne virus monitoring solution. b) Our prototype EC bioassay platform consisting of several components and accessories: air sampling device for the enrichment of particulate SARS‐CoV‐2 virus; magnetic‐bead embedded chip for real‐time SARS‐CoV‐2 virus lysis, RNA extraction and reverse transcriptase, and DNA isolation; continuous‐flow PCR microfluidic chip for the amplification of SARS‐CoV‐2 target genes; enzyme cleavage assisted EC bioassay for the identification and quantitation of SARS‐CoV‐2 bioaerosols (diagrams created with BioRender.com). c) HCoV‐229E virus was cultured in our lab (biosafety level 2) and used to evaluate the performance of our prototype sensing platform. d) qPCR data profile concerning the DNA of cultivated HCoV‐229E virus, which was extracted from the magnetic‐bead embedded microfluidic chip, indicating the successful isolation of the HCoV‐229E nucleotides. e) The temperature pattern of the continuous‐flow PCR chip captured by a thermal infrared camera. The heat distribution was consistent with the temperatures required for dsDNA denaturation, primer annealing, and genome extension. f) Fluorescence (FL) intensity (SYBR green as the indicator) of 0, 10, 20, and 30 cycles amplification of HCoV‐229E DNA, demonstrating the continuous‐flow PCR microfluidic chip was capable of amplifying the cDNA of coronavirus.

The spread of SARS‐CoV‐2 is thought to be mainly transmitted by respiratory droplets coming from an infected human, leading to viruses in the air (**Figure** **1**a).^[^ ^9^ ^]^ We sampled the SARS‐CoV‐2 and HCoV‐229E bioaerosols by filter‐based cassettes (Figure S1, Supporting Information), eluted virus‐laden particles into liquid, and conducted the virus lysis, RNA extraction and cDNA synthesis, continuous‐flow PCR amplification, and EC bioassay detection (Figure 1b). The cultivated HCoV‐229E virus was used as a model coronavirus to evaluate our prototype platform (Figure 1c). The sampled particles were incubated in the lysis buffer, and the virus‐containing eluent was then transferred to the microfluidic chip (Figure S2, Supporting Information). The RNA of the collected virus was selectively bound to the surface of the magnetic beads while other contaminants (e.g., proteins) stayed in the solution. Purified RNA was transcribed into its complementary DNA (cDNA) via reverse transcription. The RT‐qPCR data profiles confirmed that the cDNA of the HCoV‐229E virus was successfully isolated in the chip (Figure 1d).

The DNA amplification was performed in a continuous‐flow PCR microfluidic chip (Figure 1b). The isolated DNA was flown through a narrow serpentine channel composed of different thermostatic zones (95, 55, and 72 °C) with the length necessary for double‐stranded DNA (dsDNA) melting, primer annealing, and genome extension. The temperature zones were in a high‐middle‐low configuration (Figure 1e; Figure S3, Supporting Information). This design kept the PCR mixture continuously flowing and reacting, allowing for a high‐throughput and real‐time preparation of specific and ample sensing targets from airborne particles. Bubble generation inside a heated microchannel could affect or even block the flow. We proposed a bubble‐free layout to eliminate the bubble formation by connecting a 0.25 mm‐inradius resist PEEK tubing to the PCR chip's outlet (1 mm‐inradius tubing). The PCR buffer was sped up in the resist tubing to maintain a constant volume flow rate, leading to a higher pressure in the serpentine channel than that in the resist tubing, thus circumventing the bubble generation. With this layout, the continuous‐flow PCR chip successfully amplified the isolated HCoV‐229E cDNA (Figure 1f).

### 2.2 Dynamics of EC Bioassay Response

**Figure 2:**
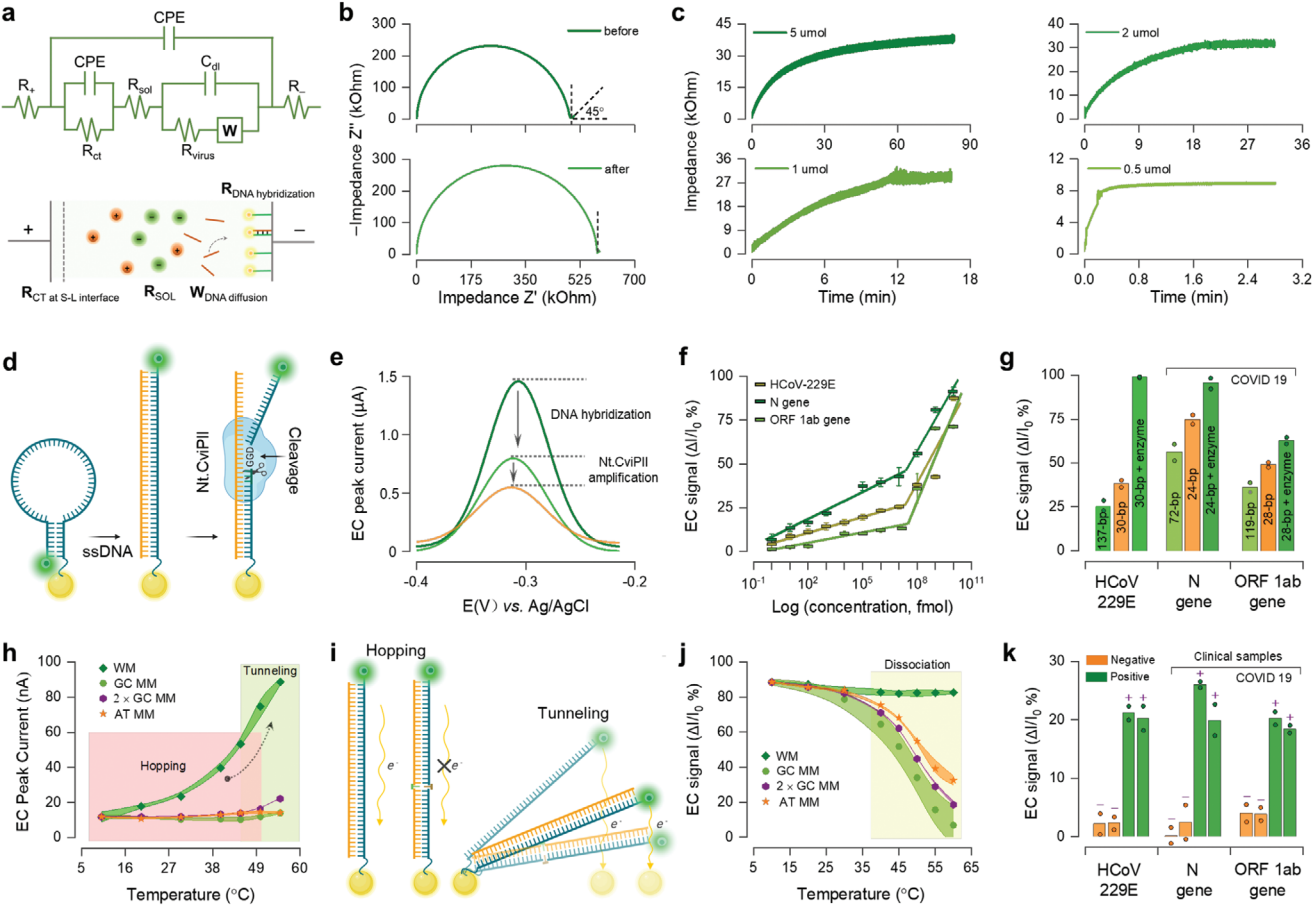
Enzymatically enhanced EC bioassay for HCoV‐229E and SARS‐CoV‐2 RNA detection. a) Randles circuit model and schematic diagram of mass transport and biorecognition events between working and counter electrodes. b) Nyquist plot of probe‐target interaction (2 µmol HCoV‐229E probes assembled electrode incubating in 1 µmol corresponding cDNA). c) Binding kinetic curves of DNA hybridization obtained by measuring the changes of *R*_virus_ over time until reaching their steady‐state regime (2 µmol HCoV‐229E probes modified electrodes incubating in 0.5, 1, 2, and 5 µmol corresponding cDNA). The impedance changes were 37.2, 31.4, 28.3, and 8.9 kOhm corresponding to 5, 2, 1, and 0.5 µmol conditions, respectively. d) Principle of endonuclease cleavage enhanced electrochemical DNA sensor. The diagram was created on BioRender.com. e) SWV peak current acquired from the hybridization‐ and cleavage‐induced signals (2 µmol HCoV‐229E probe modified electrode incubating in 100 nmol corresponding cDNA). f) Calibration curves of the change of SWV peak current (ΔI/I_0_) versus logarithmic concentration of the HCoV‐229E cDNA, the N and ORF 1ab genes of SARS‐CoV‐2 in a range of concentrations from 1 fmol to 10 µmol. The target sequences were in the length of 137 base pair (bp) for HCoV‐229E sequences, 72‐bp for N genes, and 119‐bp for ORF 1ab genes of SARS‐CoV‐2 virus, consisting of their forward and reverse primers (Table S2, Supporting Information). g) Current response to the hybridization of the probe with that of perfect cDNA (light‐green bar, length of cDNA was equal to that of probe) and RT‐qPCR equivalent cDNA (orange bar, herein cDNA including the fragments of its forward and reverse primers), and enzyme cleavage induced conformation change of dsDNA (green bar) (2 µmol HCoV‐229E probes anchored electrodes incubating in 100 nmol corresponding cDNA). h) Peak current versus elevated temperature for well‐match, single (GC and AT) and double (2 × GC) nucleotides mismatch (2 µmol HCoV‐229E probes anchored electrodes incubating in 100 nmol their cDNA buffers). i) Illustration of electron transfer from the endpoint of dsDNA or ssDNA to the sensing electrode via tunneling‐hopping regime, either by coherent tunneling over a short distance or incoherent hopping over a long distance. j) Specificity evaluation of the EC bioassay with HCoV‐229E genes, tested with well‐matched and mismatched cDNA. Different from the current acquired at an elevated temperature in (h), the SWV curve was recorded at 25 °C. k) Clinical validation of analytical concordance between the developed EC bioassay and RT‐qPCR. The symbol of “‘+”’ refers to the RT‐qPCR confirmed positive samples, and “‘−”’ stands for the RT‐qPCR verified negative samples. The error bars concerning the data points were connected in B‐spline style, and the color‐filled area represented the deviation magnitude.

Time‐ and concentration‐dependent dynamics of surface‐mediated DNA biosensing were associated with the probe packaging density, affecting EC bioassay's sensitivity and response time.^[^ ^10^ ^]^ We established a phase‐sensitive technique based on the impedance measurement that can probe the sensing electrode's dielectric properties in a non‐destructive way, allowing for monitoring the dynamic process of cDNA detection. The chips for binding kinetic studies were prepared by incubating gold‐nanoparticle‐modified screen‐printed electrode (AuNPs‐SPE) in HCoV‐229E probe solutions with various concentrations, forming different surface‐anchoring densities (Experimental Section; Figure S4, Supporting Information). In **Figure** **2**a, Randles circuit model and schematic diagram illustrated mass transport and biorecognition process for cDNA detection, converting surface biorecognition events to an impedance signal.^[^ ^11^ ^]^ Before testing, the impedance system was double‐checked to exclude unstable connections and potential interferences (Figure S5, Supporting Information). After probe hybridizing with its cDNA, *R*_virus_ increased from 497.69 to 581.46 kOhm, indicating target sequences were immobilized onto the electrode (Figure 2b). The dynamic process was depicted by real‐time measurement of *R*_virus_ at a specific frequency, that is, the low‐frequency intercept on the X‐axis in the Nyquist plot (Figure S6, Supporting Information). The 5‐µmol probe density demonstrated a significant resistance response (37.2 kOhm) while it required more than 90 min to reach its surface saturation (Figure 2c). In contrast, a 0.5‐µmol probe density needed less time (30 s) to reach the saturated condition but demonstrated a lower impedance change (8.9 kOhm). We next calculated the binding kinetic constants relying on the dynamic curves (Table S1, Supporting Information). According to achieved kinetic parameters, the 2‐µmol probe density was selected as the optimal condition for the top sensor response, and the inter‐probe distance was calculated as 3.6 nm (Experimental Section; Figure S7, Supporting Information).^[^ ^12^ ^]^ The inter‐probe spacing was responsible for surface‐mediated steric effects which can affect the probe's ability to hybridize with incoming cDNA strands, particularly capturing genes of different lengths in a complex biological matrix.^[^ ^13^ ^]^

### 2.3 EC Current Responses to HCoV‐229E and SARS‐CoV‐2 cDNA Detection

The multichannel EC bioassay platform consisted of the multichannel bioassay, a thermal‐controlling system, and the microfluidic reactor (Figure S8, Supporting Information). The bioassay was prepared by covalently immobilizing probes onto AuNPs‐SPE (Table S1 and Figure S7, Supporting Information). The probes were the HCoV‐229E or SARS‐CoV‐2 single‐stranded DNA (ssDNA) with a hairpin structure at its 5′ end and a redox reporter at the 3′ terminus (Table S2, Supporting Information). The probe allowed the target sequence to hybridize and provide the electrons to generate a redox current.^[^ ^14^ ^]^ The coronavirus detection was conducted with a two‐step protocol: hybridization of the target ssDNA and endonuclease cleavage of site‐specific nucleic acids (Figure 2d). Upon hybridizing with intended genes, target‐probe duplexes constrained the electron transfer by driving redox reporter away from the electrode, leading to the current decrease. Enzyme‐cleavage triggered a second current decrease by cutting away the redox tag, thus amplifying the signal. A clear two‐step signal response was obtained accordingly (Figure 2e). Three calibration curves were then acquired and used to quantitate airborne HCoV‐229E and SARS‐CoV‐2 viruses. Our multichannel EC bioassay exhibited an ideal signal‐concentration linear relationship in the range of concentrations from 1 fmol to 10 nmol (≥97.7% in linear regression analysis), and ultralow levels of limit‐of‐detection (1.42 fmol for HCoV‐229E, 1.65 fmol for SARS‐CoV‐2 N, and 11.3 fmol for SARS‐CoV‐2 ORF 1ab), which were critical for quantitating coronavirus genes in low doses (Figure 2f).

The length of target sequences can affect the signals, leading to inaccurate quantitation. Compared to long‐length cDNA as the sensing targets, a higher current suppression was obtained using short‐length ones (38.24%, 74.45%, and 48.92% for 30‐bp HCoV‐229E, 24‐bp N, and 28‐bp ORF 1ab gene of SARS‐CoV‐2, respectively) (Figure 2g). In the near‐surface, the target cDNA underwent a 2D search of an immobilized complement. Large sequences could intensify the molecular crowding, affecting their ability to hybridize with the probes and causing a size‐dependent current suppression.^[^ ^10^ ^]^ Thus, we used the identical sequences from the continuous‐flow PCR amplification for the calibration curve determination (Figure 2f). Endonuclease cleavage additionally increased the signal and thus improved the LoD of EC bioassay (Figure 2g).

### 2.4 Specificity Evaluation on Coronavirus cDNA Detection

Non‐specific nucleotide products from the PCR amplification and mutant sequences from the lysis buffer can inevitably adsorb onto immobilized probes, affecting the signal properties. For non‐specific DNA hybridization, once the bonded sequence is altered, the hybridized duplex's thermal stability changes, leading to different melting temperatures (*T*_m_).^[^ ^15^ ^]^ Thus, non‐specific sequences could be well‐distinguished based on the *T*_m_ of hybridized probe duplexes.^[^ ^16^ ^]^ The bioassay system was integrated with a computer‐programmed thermoelectric controller, which performed electrochemical melting curve analysis with a high temperature resolution that can discriminate the *T*_m_ between hybridized probe duplexes from specific and non‐specific sequences.

We then designed a series of mismatched (MM) sequences and used them to validate the high specificity of our EC bioassay. The sequences and their corresponding *T*_m_ were listed in Table S2, Supporting Information. We provided the results of the square wave voltammetry (SWV) peak current measured at different temperatures for the hybridized probes (Figure 2h). In the temperature range from 10 to 45 °C, no dissociation happened, and the current was assigned to the long‐range electron hopping in DNA. A higher current and more pronounced current increment with temperature were observed for well‐matched (WM) hybridized probes than MM ones (Figure 2h). As illustrated in Figure 2i, electron transfer from the endpoint of dsDNA or ssDNA to the sensing electrode was thought to be an intermediate regime between hopping and tunneling, arising from electrons either hopping through intact *π*‐stack base pairs or tunneling by contact‐mediated collision.^[^ ^17^ ^]^

The test steps to demonstrate specificity are described in Experimental Section. The signal response was recorded at 25 °C for detecting WM and MM HCoV‐229E hybridized dsDNA duplexes as a function of the elevated temperature (Figure 2j). Clear and distinguishable EC signals were obtained for the various sequences. After controlling the temperature at 60 °C, the signal at 25 °C for the WM helix was still maintained, while others decreased due to the dissociation of duplexes. The hybridized probes with one A‐T mismatch exhibited a lower current decay than that containing one G‐C mismatch.^[^ ^18^ ^]^ The hybridized probe duplexes consisting of one and two G‐C mismatched joints showed a similar decrease in signals (Figure 2j).

We further confirmed the analytical accuracy of the EC bioassay by testing COVID 19 samples, including nasopharyngeal swab samples (diagnosed as positive or negative for SARS‐CoV‐2 virus), and artificially prepared HCoV‐229E samples. EC bioassay showed a strong signal for positive samples while exhibited a weak response to negative specimens, indicating our system possessed a high specificity toward real‐world sample detection (Figure 2k).

### 2.5 Studies on Airborne Coronavirus Transmission and System LoD

**Figure 3:**
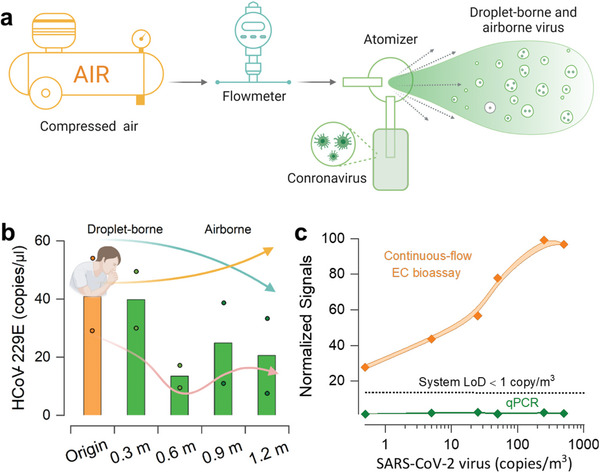
Designed experiment for simulating the airborne coronavirus transmission and LOD of the prototype sensing system. a) Test setup for generating HCoV‐229E virus in the air. The spraying was at an airflow rate of 22 L min^−1^ and maintained for 15 min (330 L), approximately equal to 66 times of the air volume flowing into or out of the lungs during a normal breath (tidal volume, V_T_). The atomizer was filled with the cultivated HCoV‐229E virus suspension, and five pieces of surgical masks were placed along with the downstream airflow (one sample every 0.3 m in the horizontal direction parallel to the ejected airflow). b) Histogram plot of the HCoV‐229E concentration, quantified by RT‐qPCR, versus different distances from the spray nozzle. Inserted annotation illustrated the intermediate droplet‐borne and airborne transmission responsible for the SARS‐CoV‐2 spread from infected individuals. c) System LoD determination. We assumed that the volume of the sampled air (m^3^) per filter followed the protocol of NABEL, the elution process was identical to the present study, and no virus was lost during the air filtering and particle elution. EC signal and qPCR responses to different concentrations of SARS‐CoV‐2 virus (in the unit of copies m^−3^) were compared. Each prepared SARS‐CoV‐2 cDNA sample was amplified with 20 cycles. The error bars concerning the data points were connected in B‐spline style, and the color‐filled area represented the deviation magnitude. The diagram was created on BioRender.com.

We provided a laboratory test to mimic the airborne transmission of coronavirus from an infected source and checked whether our sensing platform could perform detection of the airborne viruses. The laboratory‐test setup was illustrated in **Figure** **3**a. The atomizer was filled with HCoV‐229E virus buffer. The suspension was sprayed at an airflow rate of 22 L min^−1^ and maintained for 1.5 min (33 l), approximately equal to 66 times of the air volume flowing into or out of the lung during a normal breath. The ejected droplets settled down and were collected by surgical masks. The HCoV‐229E RNA was isolated and reverse transcribed into its cDNA by our integrated microfluidics. The enriched cDNA was then amplified in the continuous‐flow PCR chip. As the reference, RT‐qPCR was used to quantitate the concentration of aerosolized HcoV‐229E at several distances away from the spray nozzle (Figure 3b). A high concentration at 0.3 m was obtained as 40 copies µL^−1^ in reverse‐transcribed solution. The concentrations at 0.6, 0.9, and 1.2 m were 13, 25, and 26 copies µL^−1^, respectively. The general trend was that the further the distance, the lower the virus concentration.

The SARS‐CoV‐2 detection in the urban ambient air requires our platform to probe low doses of viruses. We conducted the SARS‐CoV‐2 cDNA (N region) detection for a series of gene concentrations with 20‐cycle amplification to determine the system LoD. The LoD was 0.1 copy µL^−1^ and can be further improved by increasing the amplification cycles. With the assumptions that the volume of the sampled air (m^3^) per filter followed the protocol of the Swiss National Air Pollution Monitoring Network (NABEL), the elution process was identical to the present study and no virus was lost during air filtering and particle elution (Experimental Section), our system could discern less than 1 copy of SARS‐CoV‐2 virus in 1 m^3^ of collected air.^[^ ^19^ ^]^ In comparison, a clear EC signal was obtained for the prepared SARS‐CoV‐2 cDNA samples, while no fluorescence signal change was detected by a spectrometer (Figure 3c).

### 2.6 Identification and Quantitation of Airborne HCoV‐229E and SARS‐CoV‐2 Viruses

**Figure 4:**
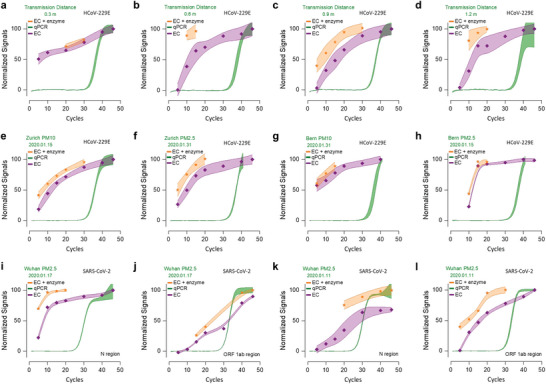
Quantitation of HCoV‐229E and SARS‐CoV‐2 in the air using enzymatically enhanced EC bioassay, with example PM samples from Bern, Zurich, and Wuhan. Signals responses to qPCR (green curves), EC bioassay (purple curves), and enzyme cleavage (yellow curves) for the detection of amplified genes from airborne coronavirus. The signals were in the form of normalized values to facilitate a comparison of their detection performances. The Nt.CviPII buffer was added to the reactor when the EC signal was higher than 8%. Thus, the data points acquired from enzyme amplification were not the same among different sample tests. a–d) Validation of analytical concordance for the quantitation of aerosolized HCoV‐229E virus between qPCR and EC bioassay. e–h) Detection of HCoV‐229E bioaerosols (PM10 and PM2.5) in residential (Bern) and road‐side (Zurich) areas during January 2020, samples provided by Swiss National Air Pollution Monitoring Network (NABEL). i–l) One‐pot dual detection of N and ORF 1ab genes of airborne SARS‐CoV‐2 virus (PM2.5) in Wuhan just before the lockdown, air‐sampling with the help of Institute of Atmospheric Physics, Chinese Academy of Science. A clear EC signal was obtained within 10‐cycle amplification concerning all tested airborne samples, indicating that our developed system could measure airborne coronavirus reliably. The error bars concerning the data points were connected in B‐spline style, and the color‐filled area represented the deviation magnitude.

The signals for aerosolized HCoV‐229E virus were recorded as a function of amplification cycles at different distances from the spray nozzle (**Figure** **4**a–d). The concentrations quantitated by the EC bioassay and RT‐qPCR were of similar magnitude and in a good correlation (Table S3, Supporting Information). Clear current responses were obtained after 10‐cycle amplification for all tested samples, while reliable RT‐qPCR signals appeared at ≈30 cycles. Enzyme cleavage further amplified the signals.

We next exploited the analytical sensitivity of the prototype system for quantitating real‐world airborne HCoV‐229E and SARS‐CoV‐2 particles. Similar signals were obtained concerning the detection of HCoV‐229E bioaerosols in PM10 and PM2.5 (particles with aerodynamic diameters less than 2.5 and 10 µm, respectively) from road‐side (Bern) and residential (Zurich) areas during January 2020 (Figure 4e–h). The concentration was quantitated according to the calibration curves and then converted to the unit of copies m^−3^ considering air sampling volume (Experimental Section). The calculated concentrations of airborne HCoV‐229E virus were 50 194 (Zurich PM10), 83 289 (Zurich PM2.5), 54 232 (Bern PM10), and 7583 (Bern PM2.5) copies m^−3^. The concentrations (N genes) were calculated as 1197 and 23 copies m^−3^ (Wuhan, PM2.5) on the sampling dates of 11 and 17 January 2020, respectively (Figure 4i–l). The obtained concentration of SARS‐CoV‐2 was higher than published data (circa 2 copies m^−3^) measured in Wuhan's public areas during February. The decrease from January to February was due to lockdown and quarantine in Wuhan (promulgated on 23 January 2020).

The lockdown policy severely limited outdoor activities, and Wuhan citizens were only allowed to leave their homes every second day for a maximum of 30 min, significantly reducing the virus emission in urban ambient air. On 13 January, China CDC (Center for Disease Control and Prevention) emergency response level was upgraded to the highest level, and strict measures were activated in Wuhan. These imposed steps curbed the spread of the virus in the air. Therefore, a lower number of airborne virus copies (23 copies m^−3^) were measured on 17 January compared with that (1197 copies m^−3^) on 11 January. Moreover, the weather on 11 January was foggy, while it was sunny and overcast on 17 January (https://www.timeanddate.com/weather/china/wuhan). The less ideal atmospheric dispersion might have contributed to the higher airborne SARS‐CoV‐2 concentration on the foggy day.

## 3 Conclusion

Few studies have reported the development of bioanalytic systems for quantitating SARS‐CoV‐2 bioaerosols in the urban area. We present a compact, highly specific bioanalysis platform based on a continuous‐flow PCR integrated multichannel EC bioassay, which exhibited an extremely low LoD and can directly detect and quantify HCoV‐229E and SARS‐CoV‐2 bioaerosols. Our system demonstrates several outstanding characteristics in terms of size, LoD, response time, specificity, etc. (Table S3, Supporting Information). Table S3, Supporting Information, highlights several advantages of our sensing platform compared to published COVID‐19 detection techniques. In contrast to those spectroscopy‐based devices for the SARS‐CoV‐2 virus detection, our EC sensing platform benefits from its competitive advantages of being portable and requiring minimum accessories for its operation, which can be easily installed in public settings. Apart from the Illumina‐ (https://emea.illumina.com) and Nanopore‐based DNA sequencing devices and PCR‐based instruments (RT‐qPCR and ddPCR), this work, for the first time, presented an alternative and facile system to quantitate both airborne and clinical SARS‐CoV‐2 specimens.^[^ ^20^ ^]^ We achieved the quantitation based on the excellent linear relationship between EC signals and logarithmic concentrations of HCoV‐229E and SARS‐CoV‐2 cDNA. The concentration determined from our EC bioassay was of similar magnitude and in good correlation with the values obtained from the RT‐qPCR (Table S4, Supporting Information), indicating our EC bioassay could quantitate the airborne coronavirus reliably. Thanks to the continuous‐flow PCR amplification step, proper probe design, and probe density optimization, the EC bioassay system exhibited an ultralow LoD (0.1 copy µL^−1^), which could discern less than 1 copy of SARS‐CoV‐2 virus in 1 m^3^ of collected air, surpassing almost all reported techniques. The excellent LoD enabled the system to quantitate the low environmental viral load of HCoV‐229E and SARS‐CoV‐2. The response‐time of fluorescence‐based methods is commonly more than 30 min, such as RT‐qPCR,^[^ ^19^ ^]^ ddPCR, recombinase polymerase amplification (RPA)^[^ ^21^ ^]^ reverse transcription‐loop‐mediated isothermal amplification (RT‐LAMP)^[^ ^22^ ^]^ and clustered regularly interspaced short palindrome repeats (CRISPR).^[^ ^23^ ^]^ In comparison, our system possesses a turnaround time of less than 15 min.

Our integrated continuous‐flow PCR chips allowed for near‐real‐time, high‐throughput, and continuous genetic isolation and amplification of HCoV‐229E and SARS‐CoV‐2 viruses, enabling on‐site preparation of specific and ample sensing targets from the collected airborne particles. In addition, our device was able to perform electrochemical melting curve analysis that can discriminate non‐specific targets from the PCR products and airborne specimens, achieving an enhanced sensor selectivity.

The clinical COVID‐19 test further confirmed the specificity of our sensor. We provided the laboratory test using cultivated HCoV‐229E virus to simulate the airborne transmission of COVID‐19 and validated that our EC platform can quantitate airborne viruses and reveal the transmission characteristics directly. With our prototype platform, we conducted the quantitation of real‐world HCoV‐229E and SARS‐CoV‐2 in airborne PM samples collected from road‐side and residential areas in Bern, Zurich, and Wuhan, with resultant concentrations verified by standard RT‐qPCR.

The ongoing COVID‐19 pandemic is exacerbated by more infective variants.^[^ ^24^ ^]^ Our continuous‐flow PCR integrated EC bioassay can be easily reconfigured within days to quantitate airborne SARS‐CoV‐2 mutants. Our technique holds a promising prospect for on‐site airborne COVID‐19 monitoring in public areas, facilitating evidence‐based control measures and early warning for infection risks.

## 4 Experimental Section

### Chemicals, Reagents, and Instrumentation

The chemicals were provided by commercial suppliers and used without purification. DNase/RNase Free water was adopted to dilute the chemicals, fetal bovine serum (FBS) for HCoV‐229E cultivation, and the SuperScriptTM III SuperMix for RNA reverse transcriptase (Thermo Fisher, USA). The QIAamp Mini Kits were selected to isolate viral RNA in clinical and environmental HCoV‐229E and SARS‐CoV‐2 samples (QIAGEN, Hilden, Germany). SYBR Green Supermix (DNA amplification reagent) was purchased from Bio‐Rad Laboratories (USA). Human coronaviruses 229E (NCPV2008101v), MRC‐5 pd19 cells (a continuous line of human lung fibroblast cell), Carbapenemase‐producing Enterobacteriaceae (CPE, Gram‐negative bacteria) were obtained from the European Collection of Authenticated Cell Cultures (ECACC). Nt.CviPII and 1× rCutSmart Buffer were provided by New England Biolabs (NEB, UK). Sulfuric acid (≥99.99%), sodium chloride (ACS reagent, ≥99.0%), chloroauric acid (≈50% Au basis), tris(2‐carboxyethyl)phosphine (TCEP), 6‐Mercapto‐1‐hexanol (MCH, ≥99%), and 1× PBS buffer were ordered from Sigma‐Aldrich (Merck, USA). The designed primers, target sequences, and sensing probes were synthesized and provided by Microsynth (Balgach, Switzerland). ChipGenie edition P was a compact, versatile instrument for on‐chip magnetic bead handling and heating, allowing for on‐chip HCoV‐229E and SARS‐CoV‐2 virus treatment, that is, cell lysis, RNA purification and reverse transcriptase, DNA isolation (microfluidic ChipShop, German). The quality and quantity of the extracted RNA and DNA were determined by gel electrophoresis and an Infinite 200 PRO plate reader (TECAN, Switzerland). CFX Connect Real‐Time PCR system was employed to detect multiplex real‐time PCR reactions (New England Biolabs, UK). Thermoelectric Peltier Modules (Mouser Electronics, USA) combined with TEC precision Peltier controller (Meerstetter Engineering, Switzerland) performed the sensing measurement. SP‐300 potentiostat was used to collect the amperometric response (BioLogic Sciences Instruments, Germany). MFIA Impedance Analyzer was a state‐of‐the‐art scientific grade instrument featuring a frequency range from 1 mHz to 5 MHz that provided real‐time impedance data, capable of characterizing the kinetics of DNA hybridization (Zurich Instruments, Switzerland).

### HCoV‐229E Cultivation, Designs of Primers and Probes

Human coronaviruses 229E was provided from the European Collection of Authenticated Cell Cultures (ECACC). Protocol for HCoV‐229E virus inoculation: MRC‐5 cells were cultured in MEM medium (Cat. 41 090 028, Thermo Fisher Scientific) with 10% FBS (Cat. 10 270 106, Thermo Fisher Scientific) and 100 U mL^−1^ Penicillin‐Streptomycin (Cat. 15 140 122, Thermo Fisher Scientific) until 90% confluence in 75 cm^2^ flask (Cat. 90 076, TPP Techno Plastic Products AG, Switzerland). Then, MRC cells were washed twice with PBS and incubated with1000 µL virus suspension at 33 °C for 2 h followed by topping up with 10 mL maintenance medium (0% FBS, 100 U mL^−1^ Penicillin‐Streptomycin) and incubating at 33 °C; after 3–4 days of incubation, freeze‐thaw for 3 times of the flask and centrifuged at 3000 rpm for 10 min, the supernatant was collected for RNA extraction, and Viral RNA was isolated by using a QIAamp viral RNA kit (QIAGEN, Hilden, Germany). Protocol for TCID_50_ (50% tissue culture infectious dose per milliliter): the day before preparing 96‐well with MRC‐5, seeding the plate with 20 000 cells per well (200 µL well^−1^ of a solution containing ≈10^5^ cells mL^−1^); 10% serial dilutions of virus suspension using cell culture maintenance medium (0% FBS) in a new 96‐well plate (virus plate) (same concentration of virus in each column); discarding cell culture medium (10% FBS) of the cell plate, washing twice with PBS buffer (200 µL well^−1^); adding 100 µL well^−1^ of virus suspension in the plate, shaking and incubation at 33 °C for 2 h; discarding virus suspension and adding 200 µL well^−1^ cell culture maintenance medium (0% FBS), incubating at 33 °C until the negative control appearing the CPE; calculatingTCID_50_ using Reed–Muench. Protocol for crystal violet staining: discarding cell culture maintenance medium (0% FBS), washing cells twice with HBSS (Hanks’ Balanced Salt Solution) 200 µL well^−1^; adding 100 µL well^−1^ 0.1% crystal violet and incubating for 3 min; discarding the crystal violet and washing cells twice with HBSS 200 µL well^−1^ and inspected with a microscope. The primers and probes concerning the ORF 1ab genomes of SARS‐CoV‐2 were designed based on the Chinese Center for Disease Control and Prevention (China CDC) recommendation. The sequences regarding the N region of SARS‐CoV‐2 were chosen based on the United States Centers for Disease Control and Prevention (U.S. CDC). The primers and probes were selected for HCoV‐229E using Primer Express Software (Perkin‐Elmer Applied Biosystems) and were based on highly conserved genomic regions. All sequences used, including probe sequences for PCR, electrochemical test, and designed mismatches, are listed in Table S2, Supporting Information.

### Experiment for Simulating the Transmission of SARS‐CoV‐2 Bioaerosols

The viruses can be transmitted by both droplets and aerosols in a close interaction scheme and by aerosols in the long‐distance propagation scenario. The artificial test platform encompassed an air compressor supplier, the pressure‐reducing valves, a digital flowmeter, and a glass atomizer. The atomizer was filled with the cultivated HCoV‐229E virus suspension, and aerosolized matters were collected with surgical face masks. The spraying was at an airflow rate of 22 L min^−1^ and maintained for 1.5 min (33 l), approximately equal to 66 times of the volume from air moved into or out of the lungs during a normal breath (tidal volume, V_T_). The mask collectors were located downstream of the airflow parallel to the spray direction, capable of mimicking the distance‐related dose (0, 0.3, 0.6, 0.9, and, 1.2 m) of coronavirus droplets and aerosols. The artificial experiment was conducted in a closed biosafety bench (Biosafety level 2 Lab).

### HCoV‐229E and SARS‐CoV‐2 Bioaerosols Sampling

The HCoV‐229E virus bounded PM10 and PM2.5 samples in Bern and Zurich residential and street regions during January 2020 were collected through the Swiss National Air Pollution Monitoring Network (NABEL). NABEL measured air pollution at 16 locations in Switzerland. The stations were distributed throughout the country and monitor pollution at representative locations (e.g., city‐center streets, residential areas, rural environment). The filter area was 153.86 cm^2^, and the total sampled air volume each day was 720 m^3^. The SARS‐CoV‐2 bioaerosols samples were supplied by the Institute of Atmospheric Physics, Chinese Academy of Science (IAP, CAS), and the airborne particles were collected just several days before the lockdown (23.01.2020) of Wuhan city. All the filter samples were stored at minus 80 °C conditions before further treatment. The sampling volume was 1627.2 m^3^ (1.13 m^3^ min^−1^, 24 h), and the filter area was 414 cm^2^. This work took out 34.44 cm^2^ (Bern and Zurich samples, corresponding to 161 m^3^ sampled air) and 4.52 cm^2^ (Wuhan samples, corresponding to 17.77 m^3^ sampled air) sections of the filters for the test and incubated them in PBS buffer solution (3 mL for NABEL specimens and 1.5 mL for Wuhan samples, respectively), shaking for 2 h to eluate the virus bounded particles. A 140 µL eluant buffer was used for the RNA extraction and the isolated RNA was 60 µL. 8 µL RNA buffer was taken for the DNA synthesis, and the reverse‐transcribed DNA solution was 20 µL. 2 µL of DNA was then used for the continuous‐flow PCR amplification.

### Patient Clinical Sample Collection

The clinical samples were confirmed with qPCR and then used for the evaluation of sensor selectivity. Clinical nasopharyngeal swabs diagnosed as positive and negative COVID 19 were from University Hospital Zurich. The samples were denatured before being transported to ETH Zurich (Biosafety level 2 Lab). The RNA purification, reverse transcriptase, and DNA isolation were implemented following the QIAamp Mini Kits’ instructions.

### Continuous Flow and On‐Chip DNA Isolation and Amplification

QIAamp Mini Kits was used to purify and isolate the viral RNA from the eluant mentioned above. The sample pretreatment was implemented in the microfluidic chip, a one‐pot bioreactor for on‐chip SARS‐CoV‐2 virus preparation steps such as lysis and DNA isolation. The experiment processes strictly followed the instructions (QIAamp Viral RNA Handbook, https://www.qiagen.com), particularly taking care of each step's incubation temperature and time. Instead of using a spin silicon membrane column, RNA purification and reverse transcriptase were implemented in a ChipGenie edition P device that is an instrument for on‐chip sample preparation steps like DNA extraction or cell lysis (Figure S2, Supporting Information). The reactor featured a click–in holder frame and contained a linearly moving magnet and temperature control. The heating element and the permanent magnet were located underneath the microfluidic chip. The heating element, as well as the permanent magnet, was located underneath the chip. The DNA was absorbed onto the magnetic beads at the last step. The DNA amplification was taken place in a continuous‐flow PCR microfluidic chip. The width and height of the microchannel were 0.5 and 0.2 mm, respectively. Before flowing into the PCR area, an additional microfluidic chamber was designed to denature other proteins (95 °C) from the commercial DNA isolation kit. The length associated with the region of DNA denaturation (95 °C) and primer annealing (55 °C) was 50 mm, and that of genome extension (72 °C) was 75 mm. The flow rate was 10 µL min^−1^ and the residence time for the buffer in the zones of dsDNA melting, primer annealing, and genome extension was 30, 30, and 45 s, respectively. An 0.25 mm‐diameter resist PEEK tubing was connected at the PCR chip's outlet (1 mm in tubing diameter) to suppress the gas bubble generation. A proportional–integral–derivative (PID) thermal controller drove the metal block heater, providing accurate and stable thermostatic zones for PCR reaction. An air gap of 3 mm was configured between each temperature zone to avoid the temperature zones overlapping.

### EC Bioassay Preparation and Test Setup Configuration

EC bioassay consisted of one sensing electrode array and a microfluidic multi‐channel (Figure S8, Supporting Information). The sensing electrode array was prepared by assembling an electrochemical DNA probe onto gold nanoparticles modified screen‐printed electrodes (AuNPs‐SPEs). The SPEs were manufactured by sequentially printing silver, silver/silver chloride (60/40), and carbon inks on electrical inert polyethylene terephthalate (PET) substrate, followed by insulating the unreactive area with the nonconductive photosensitive paste, featuring the silver/silver chloride reference electrode, carbon working and auxiliary electrodes. The oxygen plasma then treated the SPEs to remove surface oxidates and clear mineral residues. Subsequently, the electroplating solution was dispensed on the SPEs surface of interest. Electrodeposition of gold nanoparticles on carbon working electrode was performed in 5 mm HAuCl_4_ containing 0.5 m H_2_SO_4_ with potentiostatic method at −0.2 V for 90 s. 50 µL of 2 µm thiolated MB‐modified DNA probe was mixed with 2 µL of 5 mm TCEP, incubating for 60 min at 25 °C to reduce the disulfide bond entirely. The reduction of the disulfide bond of the probe DNA was complete within 60 min as evidenced by the loss of the blue color of MB^[^ ^25^ ^]^ Next, 20 µL of 2 µmol DNA probes were dispensed on the working electrode, incubated for 2 h in the dark. Then the electrode was successfully immobilized with sensing probes through a 5′ thiol anchor with a redox MB reporter at the 3′ terminus. The modified electrode was cleaned with PBS buffer and incubated in 20 µL of 2 mm freshly prepared MCH solution for 2 h at room temperature in the dark, followed by rinsing with deionized (DI) water. The prepared electrodes were dried with compressed nitrogen, sealed with parafilm, and stored in the dark at −20 °C. The EC bioassay for binding kinetic studies was prepared by incubating gold‐nanoparticle‐modified screen‐printed electrodes (AuNPs‐SPEs) in HCoV‐229E probe solutions with various concentrations, forming different probe densities. A microfluidic chip (50 µL in total volume for a single chamber) was assembled on the electrodes, forming the bioassay reactor. The computer‐programmed thermoelectric controller (TEC) was integrated into the sensing reactor to drive the Peltier element, equipping with one Pt100 and NTC thermal sensor concerning monitoring the temperature on the Peltier plate and heat sink, allowing for rapid heat conduction and precise temperature control (Figure S8, Supporting Information).

### Binding Kinetics and Probe Density Studies

The biorecognition analysis started with the sensing ligand immobilizing the sensor chip surface, then adding the HCoV‐229E target analyte. A digital impedance analyzer measured the interaction of the ligand and analyte as a change in impedance over time. In Randles circuit model diagram (Figure 2a), *R*_ct_ was defined as the interfacial resistance induced by the electron transfer from a liquid electrolyte to the solid electrode. *R*_ct at S‐L interface_ referred to the resistance at the counter electrode and was considered as a constant. *R*_+_, *R*_‐,_ and *R*_sol_ referred to the resistance of two‐pin conductors and electrolytes. *R*_sol_ was related to the mobility of electrolytes and independent of biorecognition. The diagonal line with a slope of 45° in the low‐frequency region referred to the mass transport of cDNA, known as Warburg diffusion (W). Double‐layer capacitance (C_dl_) was an intrinsic feature of polarized electrodes. The constant‐phase element (CPE) described the universal non‐ideality of the electrode. The impedance data could be interpreted and analyzed by either Nyquist or Bode plots. In the Nyquist plot, the high‐frequency intercept on the X‐axis referred to the electrolyte ohmic resistance, while the value of the low‐frequency intercept reflected the charge‐transfer resistance at the sensing electrode interface. Electron transfer resistance obtained from the surface of the working electrode (*R*_virus_) quantified the electrostatic or steric barrier adjacent to the surface of the SARS‐CoV‐2 probe anchored electrode, which was associated with the DNA hybridization (*R*_DNA hybridization_). The binding kinetic constant of DNA hybridization, **K** (mol^−1^), can quantitatively describe the steady‐state of biorecognition, defined as the dissociation constant (**K_d_**, mol^−1^ s^−1^) divided by the association constant (**K_a_**, mol^−1^ s^−1^) (Table S1, Supporting Information). The binding model was based on Langmuir's theory, which describes a 1:1 biorecognition where one ssDNA ligand interacts with one analyte ssDNA^[^ ^26^ ^]^ Complex formation followed pseudo‐first‐order kinetics, and it was assumed that the binding is equivalent and independent for all binding sites^[^ ^27^ ^]^ For performing equilibrium analysis, the time‐series impedance data were used, in which the impedance signal already approached the enduring value, and the responses of DNA hybridization reached its equilibrium, to fit the model equations and obtain corresponding constant values (http://www.labfit.net/). The binding kinetics of DNA hybridization was associated with the probe's packing density, affecting sensor sensitivity. The probe density, that is, the number of sensing DNA probe moles per unit electrochemical active surface area (ECSA) of the electrode, can be calculated based on a Nernstian electron source model, which was a well‐established relationship for the alternating current voltammetry (ACV) of a reversible surface monolayer assembly (SAM) redox reaction (Figure S9, Supporting Information).^[^ ^25^ ^]^ ECSA and the roughness factor (ratio between the ECSA and the geometrical area of the electrode) were 0.16 cm^2^ and 2.25, calculated by integrating the charge associated with the reduction of gold oxide in the cyclic voltammograms recorded in 0.5 m H_2_SO_4_ at the scan rate of 100 mV s^−1^ (Figure S7, Supporting Information). Moreover, ECSA estimated that the charge required to reduce superficial gold oxides was 390 µC cm^−2^. An optimal probe density concerning a 2 µmol probe was obtained (2.07 nmol cm^−2^).

### Specificity and LoD Measurement

The probes were immobilized to the electrodes through a 5′ thiol anchor with a redox reporter (methylene blue, MB) at the 3′ terminus. The biorecognition process opened the hairpin structure of the ssDNA probe, leading to the increment of distance for electron transfer. Nt.CviPII recognized the CCG or CCA sites of hybridized dsDNA and cleaved its phosphate bonds near the cytosine, peeling off the redox probe away from the sensing electrode. Melting curve analysis assessed the thermal stability of dsDNA duplex that reflects the energy required to break the base‐base hydrogen bonds, capable of recognizing the variants such as single‐nucleotide polymorphisms (SNPs), small insertions, and deletions. The number of hydrogen bonds of the well‐matched (WM) hybridized probe duplex was higher than that of the mis‐matched (MM) hybridized duplex. The WM hybridized probes can be distinguished from those containing MM genes by elevating the reaction temperature until all MM sequences were dissociated from probes. Considering only a single hydrogen bond was reduced between G‐T and G‐C base‐pairs, it was thus challenging to differentiate the WM and single G‐T mismatch contained hybridized probe duplexes because their melting temperatures (*T*_m_) are close. The EC bioassay performed electrochemical melting‐curves measures with an ultrahigh temperature resolution to distinguish a single nucleotide mismatch. 50 µL of 2 µm DNA buffer was pumped (20 µL min^−1^) into the bioassay reactor at 25 °C for 20 min (determined from above binding kinetic studies) to conduct the biorecognition. The bioassay chip was rinsed entirely with deionized water, followed by filling the chamber with 50 µL of 0.1 mmol NaCl solution (electron and charge transfer medium). Temperature‐dependent change in SWV peak current indicated the difference in melting temperature between well‐matched and mismatched target sequences. To obtain the melting curves, the temperature of the Peltier plates was ramped from 10 to 60 °C in 5 °C increments, holding for 2 min at each temperature point. During the incubation time at each temperature point, the amperometric measurement was conducted using the square wave voltammetry (SWV) with a step potential of 10 mV, an amplitude of 25 mV, and a frequency of 25 Hz (Figure 2h). At the end of each temperature ramp, the bioassay chamber was returned to 25 °C, washed with DI water, filled with 50 µL of 0.1 mmol NaCl solution, and performed another round of SWV scans (Figure 2j). According to the current melting curve response, the critical temperature was 55 °C for the standard test that allows dissociating other non‐specific sequences. To achieve the specificity, the test steps were established as follows: setting the temperature at elevated values up to the 55 °C of the MM sequence hybridized probes, holding for 2 min for opening the hairpin structure of sensing probes and dissociating the non‐specific bindings, and flushing the chamber with 0.1 mmol NaCl solution to remove the unwanted sequences, then resetting the reactor back to 25 °C for the SWV measurement. A range of target concentrations from 0.1 fmol to 1 µmol was employed for the LoD measurement, and the test procedures followed the above test protocol. The cleavage of hybridized dsDNA was conducted in the 1‐unit Nt.CviPII dissolved 1 × rCutSmart buffer at 37 °C, holding on 30 min then rinsing with 0.1 m NaCl solution. Nt.CviPII was a nicking endonuclease that cleaves only one strand of DNA on a dsDNA substrate. The cleavage sites were located at CCG and CCA. The EC signal was defined as ΔI divided by I_0_. The detection threshold was set at three times the standard deviation of the signal from a blank. I_0_ is the initial peak current, and ΔI refers to the current change between their initial ssDNA and hybridized dsDNA conditions.

### Detection and Quantitation of HCoV‐229E and SARS‐CoV‐2 Bioaerosols

The high analytical sensitivity of the multichannel continuous‐flow EC bioassay was exploited for detecting HCoV‐229E and SARS‐CoV‐2 bioaerosols. The cell lysis, RNA extraction, and reverse transcriptase, and DNA isolation of clinical samples and environmental were implemented within the ChipGenie edition P device. The isolated HCoV‐229E and SARS‐CoV‐2 genomes were amplified through our designed continuous‐flow PCR microfluidic chip. The specifically amplified DNA was heated to 95 °C, holding for 15 min, opening the dsDNA structure, quickly quenched in the ice‐water bath to unwind the DNA duplex into dsDNA, and transferred to EC bioassay reactor for the sensor measurement. The DNA detection followed the proposed experimental protocol. The concentration of airborne coronavirus in the air (*C*_a_), in the unit of copy m^−3^, was dependent on the volume of the sampled air *V*_a_ (m^3^) and volume of the buffer liquid *V*_b_ (µL) used to elute the viruses from the collected PM within *V*_a_. No loss of viruses were assumed during the air sampling and the particle elution, then the concentration in the air (*C*_a_) could be calculated as below:

(1) $$C_{a}=\frac{C_{b}\;\times \;V_{b}}{V_{a}}\;$$

whereas *C*_b_ (copy µL^−1^) was obtained from the EC sensor. The system LoD, in the unit of copy m^−3^, was therefore dependent on *V*_a_ and *V*_b_. Larger air‐sampling volume or less solution for the elution would contribute to obtaining a lower value of system LoD.

### Statistical Analysis

Smoothed SWV and impedance curves were obtained using a three‐point adjacent averaging function. SWV peak current would shift to a negative direction concerning the measurement of the melting curve. Thus, the position and the range of SWV data sampled were kept the same as others for extracting the baseline. The normalized signals were calculated by setting the maximum SWV peak current or qPCR to a value of 100%. All the data were presented from experiments repeated at least three times unless otherwise specified. The limit of detection (LoD) was set at three times the standard deviation of the signal from a blank.

## Conflict of Interest

The authors declare no conflict of interest.

## Supporting information

Supporting Information

## Data Availability

The data that support the findings of this study are available from the corresponding author upon reasonable request.
