# Supplementary material for: Direct Quantitation of SARS‐CoV‐2 Virus in Urban Ambient Air via a Continuous‐Flow Electrochemical Bioassay
Source: Adv Sci (Weinh). 2023 May 24;10(22):2301222. doi: 10.1002/advs.202301222 (PMC10401087; doi:10.1002/advs.202301222)
Supplement: Supplementary file 1 — Supporting Information [file ADVS-10-2301222-s001.pdf]

ADVANCED SCIENCE
Open Access

Supporting Information

for *Adv. Sci.*, DOI 10.1002/advs.202301222

Direct Quantitation of SARS-CoV-2 Virus in Urban Ambient Air via a Continuous-Flow Electrochemical Bioassay

*Fuze Jiang, Bei Liu, Yang Yue, Yile Tao, Zhen Xiao, Meng Li, Zheng Ji, Jiukai Tang, Guangyu Qiu, Martin Spillmann, Junji Cao, Lianjun Zhang and Jing Wang**

Supporting Information

Direct Quantitation of SARS-CoV-2 Virus in Urban Ambient Air *via* a Continuous-Flow Electrochemical Bioassay

Fuze Jiang[a,b,+], Bei Liu[a,b,c,+], Yang Yue[a,b], Yile Tao[a,b], Zhen Xiao[d,e], Meng Li[f], Zheng Ji[a,g,h], Jiukai Tang[a,b], Guangyu Qiu[a,b], Martin Spillmann [a,b], Junji Cao[i], Lianjun Zhang[d,e], Jing Wang [a,b]*

[a] Dr. F. Jiang, B. Liu, Dr. Y. Yue, Y. Tao, Dr. Z. Ji, Dr. J. Tang, Dr. G. Qiu, Dr. M. Spillmann, Prof. J. Wang
Institute of Environmental Engineering, ETH Zürich, Zürich, CH-8049, Switzerland.
E-mail: jing.wang@ifu.baug.ethz.ch.

[b] Dr. F. Jiang, B. Liu, Dr. Y. Yue, Y. Tao, Dr. J. Tang, Dr. G. Qiu, Dr. M. Spillmann, Prof. J. Wang
Advanced Analytical Technologies, Empa, Dübendorf, CH-8600, Switzerland.

[c] B. Liu
School of Environment, Harbin Institute of Technology, Harbin, 150900, China.

[d] Z. Xiao, Prof. L. Zhang
Institute of Systems Medicine, Chinese Academy of Medical Sciences & Peking Union Medical College, Beijing, 100005, China.

[e] Z. Xiao, Prof. L. Zhang
Suzhou Institute of Systems Medicine, Suzhou, 215123, China.

[f] Dr. M. Li
Zurich Instruments AG, Zürich, CH-8005, Switzerland.

[g] Dr. Z. Ji
School of Geography and Tourism, Shaanxi Normal University, Xi'an 710119, China.

[h] Dr. Z. Ji
International Joint Research Centre of Shaanxi Province for Pollutant Exposure and Eco-Environmental Health, Xi'an, 710062, China.

[i] J. Cao
Institute of Atmospheric Physics, Chinese Academy of Sciences, Beijing, 100017, China.

[#] These authors contributed equally: Fuze Jiang, Bei Liu.

*E-mail: jing.wang@ifu.baug.ethz.ch.

Table of contents

Supporting Information Figure S1 Photograph of airborne SARS-CoV-2 virus collection by filter-based cassette.

Supporting Information Figure S2 DNA isolation of HCoV-229E and SARS-CoV-2 bioaerosols in the microfluidic.

Supporting Information Figure S3 Photograph of continuous-flow PCR microfluidic device for DNA on-site DNA amplification.

Supporting Information Figure S4 Manufacturing the HCoV-229E and SARS-CoV-2 electrochemical bioassay.

Supporting Information Figure S5 Impedance calibration with a current oscillation and frequency stability test.

Supporting Information Figure S6 Nyquist, Phase and Blode plot for the binding kinetic studies of HCoV-229E DNA hybridization.

Supporting Information Figure S7 Probe density estimation based on the Nernstian electron source model.

Supporting Information Figure S8 Photograph and schematic diagram of EC bioassay sensing platform.

Supporting Information Figure S9 CVs and SWVs characterization of sensing electrode preparation.

Supporting Information Table S1 Kinetic constants of HCoV-22E hybridization measured by EC bioassay.

Supporting Information Table S2 Primers, targets, and probes for HCoV-229E and SARS-CoV-2

RNA detection.

Supporting Information Table S3 Comparison of continuous-flow EC bioassay with other technologies for detecting SARS-CoV-2 virus from the patient and airborne samples.

Supporting Information Table S4 Validation of analytical concordance for the quantitation of aerosolized HCoV-229E virus between qPCR and EC bioassay.

References

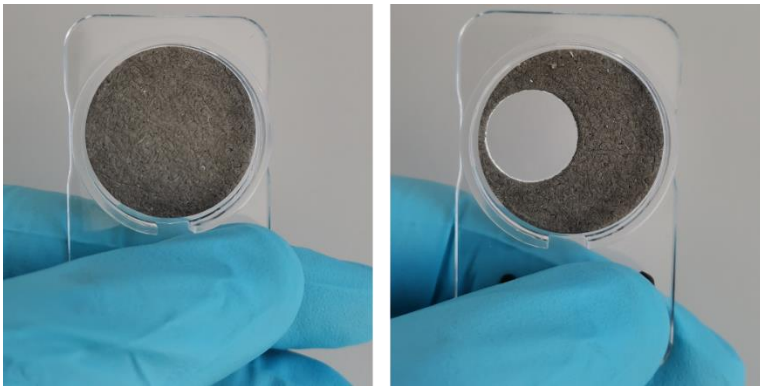

Figure S1. Photograph of airborne SARS-CoV-2 virus collection by filter-based cassettes. The SARS-CoV-2 bioaerosols samples were sampled in Wuhan city with the help of the Institute of Atmospheric Physics, Chinese Academy of Science (IAP, CAS), and the airborne particles were collected with filter-based cassettes just several days before the lockdown (21.01.2020) of Wuhan city. An area of the filter was punched out for virus detection.

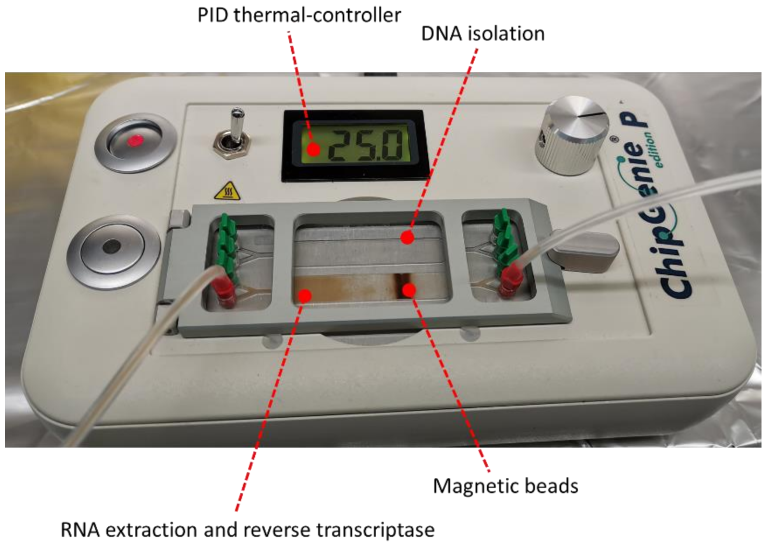

Figure S2. Photograph of custom-designed microfluidic devices for DNA on-site DNA amplification. The RNA purification and reverse transcriptase were implemented in a ChipGenie® edition P device, an instrument for on-chip biological matrices preparation steps such as DNA extraction or cell lysis. The instrument of cigar box size features a click–in holder frame and contains a linearly moving magnet and temperature control. The heating element, as well as the permanent magnet, is located underneath the chip. The DNA was absorbed onto the magnetic beads at the last step.

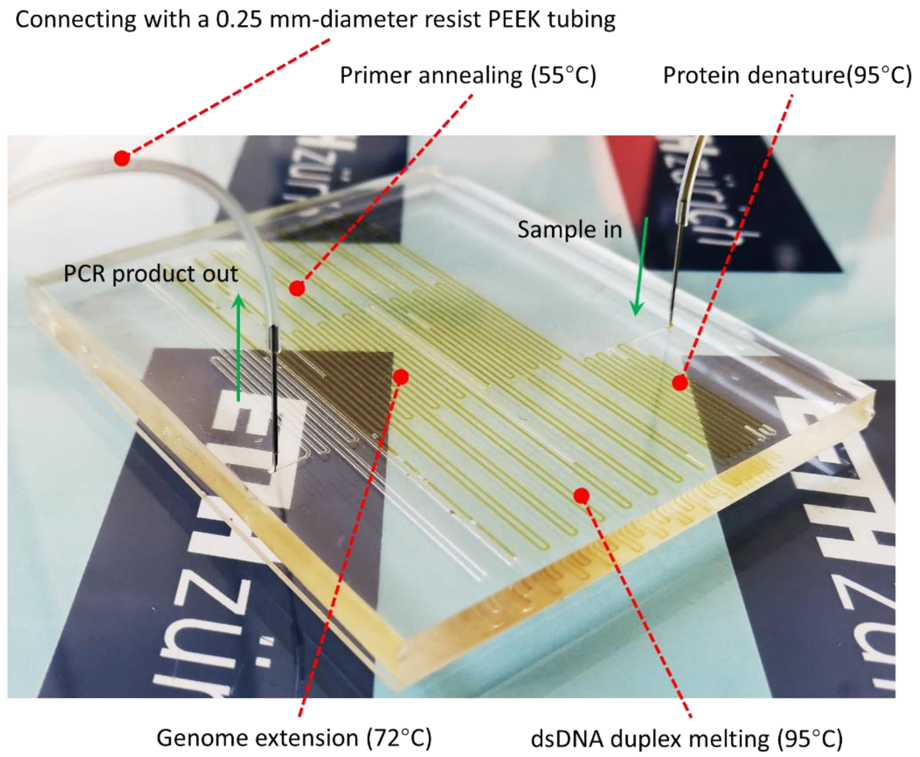

Figure S3. Continuous-flow PCR microfluidic for DNA on-site amplification. The width and height of the PCR microchannel are 0.5 mm and 0.2 mm, respectively. Before flowing into the PCR area, an additional microfluidic chamber is designed to denature other proteins (95 °C) from the commercial DNA isolation kit. The length associated with the region of DNA denaturation (95 °C) and primer annealing (55 °C) is 50 mm, and that of genome extension (72 °C) is 75 mm. The flow rate was 10 μl/min which kept the dwell time of each temperature zone at the 30 s, 30 s, and 45 s, respectively. An 0.25 mm-diameter resist PEEK tubing is connected at the outlet (1.6 mm in tubing diameter) of the PCR chip to suppress t gas bubble generation.

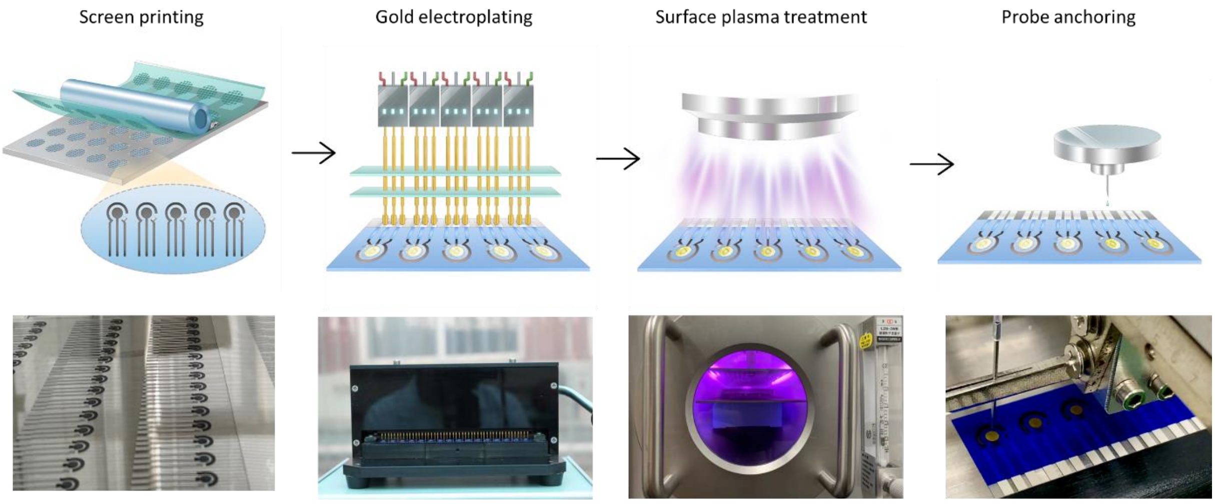

Figure S4. Manufacturing the HCoV-229E and SARS-CoV-2 electrochemical bioassay. The sensing electrode array was prepared by assembling an electrochemical DNA probe onto gold nanoparticles modified screen-printed electrodes (SPEs). The SPEs were manufactured by sequentially printing silver, silver/silver chloride (60/40), and carbon inks on electrical inert polyethylene terephthalate (PET) substrate, followed by insulating the unreactive area with the nonconductive photosensitive paste, featuring the silver/silver chloride reference electrode, carbon working and auxiliary electrodes. The oxygen plasma then treated the SPEs to remove surface oxidates and to clear mineral residues. Subsequently, the electroplating solution was dispensed on the SPEs surface of interest. Electrodeposition of gold nanoparticles on carbon working electrode was performed in 5 mM $HAuCl_4$ containing 0.5 M H_2SO_4 with potentiostatic method at -0.2 V for 90 s. DNA probes were then dispensed on the working electrode and incubated for 2 h in the dark. Then the electrode was successfully immobilized with sensing probes through a 5’ thiol anchor with a redox methylene blue (MB) reporter at the 3’ terminus.

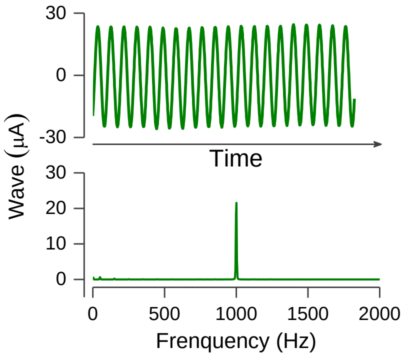

Figure S5. Impedance calibration with a current oscillation and frequency stability test. The current and the peak distribution against the applied frequency (1,000 Hz) were recorded to check the potential unstable connections and exclude potential interferences from surrounding online instruments. The current exhibited a perfect sinusoidal wave and a clear peak at the applied frequency (1,000 Hz), indicating the instrument is under a stable measurement condition. The small peak at 50 Hz is due to the commercial power frequency in Switzerland.

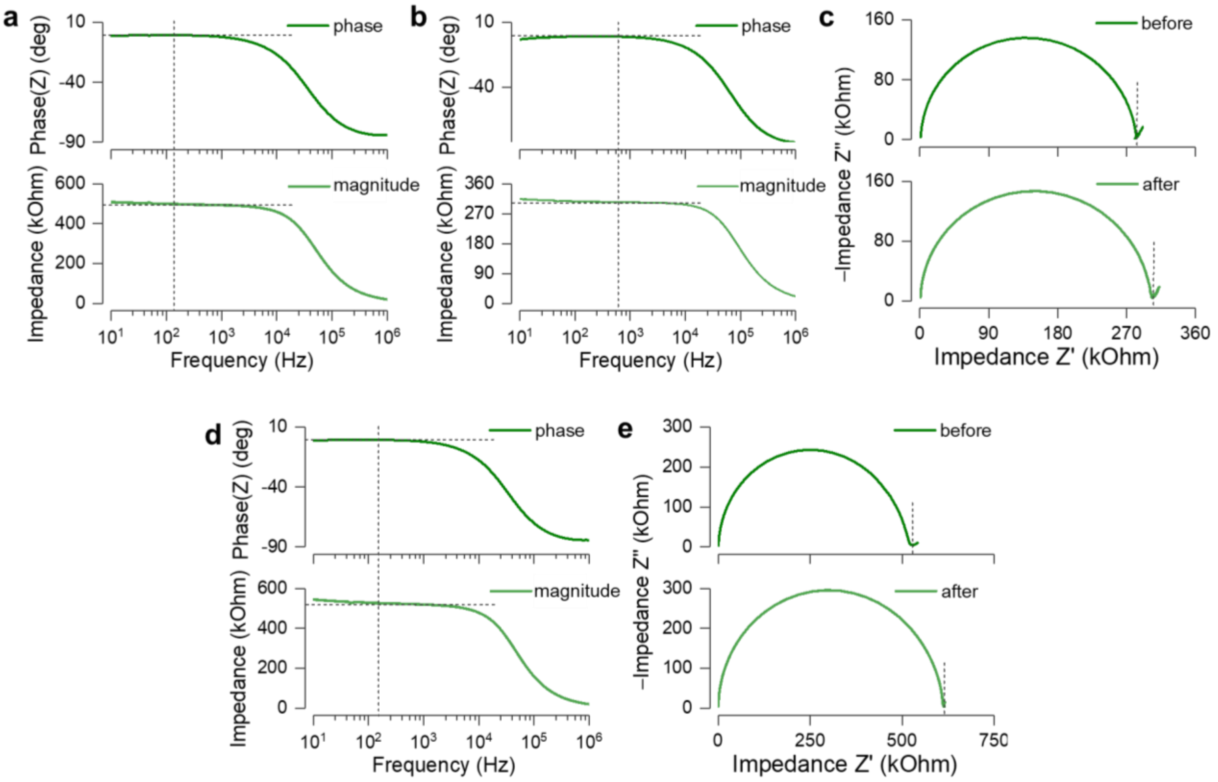

Figure S6. Nyquist, Phase, and Bode plot for the binding kinetic studies of HCoV-229E DNA hybridization. Phase and magnitude plots are the phase change graphs, commonly expressed in degrees, as a sweep of frequency in the reverse direction, illustrating an asymptotic approximation of the frequency response. Bode and magnitude plots for 2 µmol HCoV-229E probes modified electrodes incubating in 1 (**b**), 2 (**a**), and 5 (**d**) µmol their cDNA; Nyquist plot for 2 µmol HCoV-229E probes anchored electrodes incubating in 2 (**c**) and 5 (**e**) µmol their cDNA.

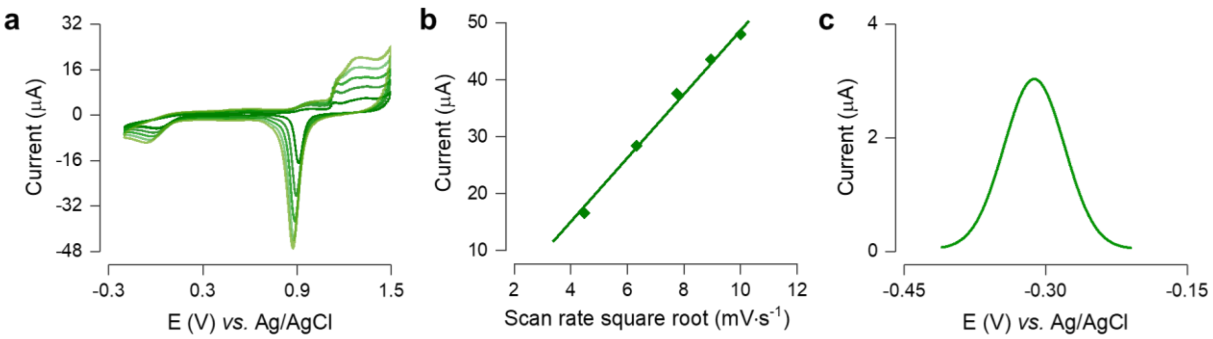

Figure S7. Probe density estimation based on the Nernstian electron source model. **a**, Reduction of gold oxide in the cyclic voltammograms (CVs) (cleaning the electrode and determining the electrochemically active surface area, EASA) recorded in 0.5 M H2SO4 at different scan rates. **b**, Plot of peak current verse the scan rate square root. **c**, Alternating current voltammetry (ACV) plot of a reversible surface redox reaction (methylene blue linked DNA probe modified working electrode).

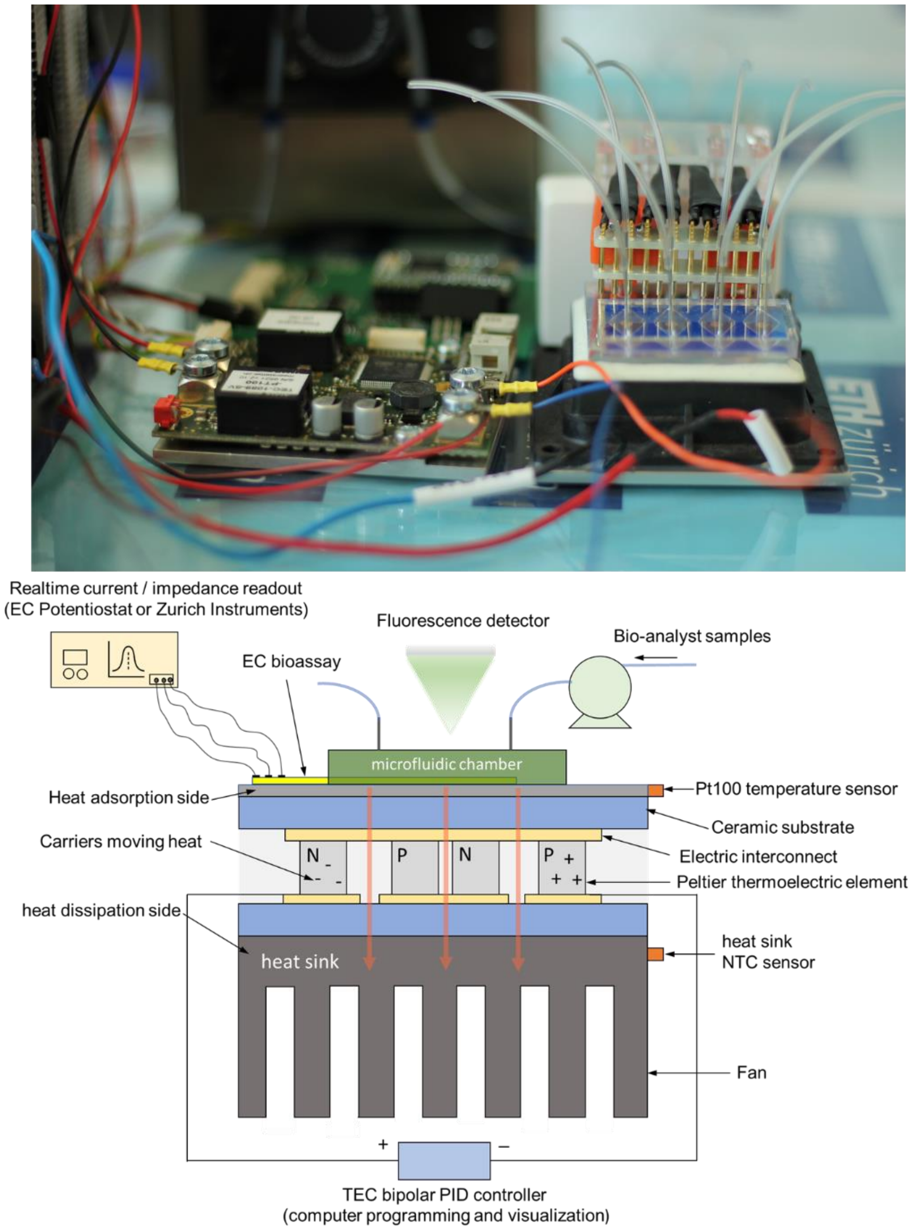

Figure S8. Photograph and schematic diagram of multichannel EC bioassay sensing platform. EC bioassay platform consists of a multichannel screen-printed electrode (SPE) sensing array, a thermal-controlling system, and a microfluidic reactor. The multichannel reactor allowed the detection and quantitation of multiple viruses simultaneously in urban ambient air. A computer-programmed thermoelectric controller (TEC) was integrated into the sensing reactor to drive the Peltier element, equipping with one Pt100 and NTC thermal sensor concerning monitoring the temperature on the Peltier plate and heat sink, allowing for rapid heat conduction and precise temperature control.

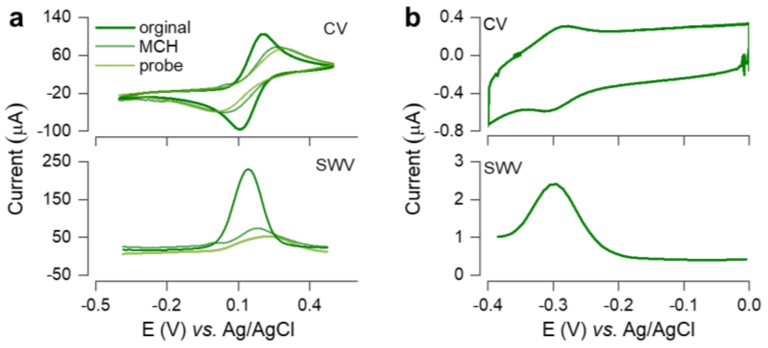

Figure S9. CVs and SWVs characterization of sensing electrode preparation. **a**, CVs and SWVs plot against original, probe anchored, and MCH modified electrodes in 50 µmol ferrocyanide/ferricyanide buffer solution. **b**, CV and SWV plot of sensing electrode in 0.1 mmol NaCl solution, the clear peak at -0.3 V associating with the redox potential of methylene blue (vs. Ag/AgCl reference electrode), indicating a sensing probe immobilized on the working electrode.

Kinetics constant	5 µmol	2 µmol	1 µmol	0.5 µmol
$\mathbf{K_a}$ ($\times 10^4$ $mol^{-1}\cdot s^{-1}$)	1.08	2.40	2.87	1.21
$\mathbf{K_d}$ ($\times 10^{-5}$ $mol^{-1}\cdot s^{-1}$)	7.97	5.37	22.77	238.83
**K** ($\times 10^8$ mol^{-1})	1.36	4.47	1.26	5.08

Table S1. Kinetic constants of HCoV-22E hybridization measured by EC bioassay. A digital impedance analyzer measured the interaction of the ligand and analyte as a change in impedance over time. From this, the dissociation ($\mathbf{K_d}$, $mol^{-1}\cdot s^{-1}$), association ($\mathbf{K_a}$, $mol^{-1}\cdot s^{-1}$), and equilibrium (**K**, mol^{-1}) constants can be derived ($\mathbf{K} = \mathbf{K_a}/\mathbf{K_d}$.). The binding model is based on Langmuir's theory, which describes a 1:1 biorecognition where one ssDNA ligand interacts with one ssDNA target. The hybridization follows the pseudo-first-order kinetics, assuming that the binding is equivalent and independent for all binding sites. For performing equilibrium analysis, we used the time-series impedance data, in which the impedance signal was already approach to flat, and the responses of DNA hybridization reached its equilibrium, to fit the model equations and obtain corresponding constant values (http://www.labfit.net/).

Name	T_m (°C)	Length (bp)	
HCoV-229E EC probe	46.5	40	5’-Thiol-GGCCC ATGAA CCTGA ACACC TGAAG CCAAT CTATG GGGCC- ATTO MB2-3’
HCoV-229E qPCR probe	16.5	30	ATGAA CCTGA ACACC TGAAG CCAAT CTATG
HCoV-229E EC target	46.7	137	CATAC TATCA ACCCA TTCAA CAAGC TCCAA CAGGC ATTAC TGTGA CCTTG CTGAG CGGCG TGCTT TACGT TGACG GACAT AGATT GGCTT CAGGT GTTCA GGTTC ATAAC CTACC TGAAT ACATG ACAGT TGCCG TG
HCoV-229E qPCR target	60.2	30	CATAG ATTGG CTTCA GGTGT TCAGG TTCAT
HCoV-229E F-primer	51.8	24	CATAC TATCA ACCCA TTCAA CAAG
HCoV-229E R-primer	53.2	20	CACGG CAACT GTCAT GTATT
SARS-CoV-2 N EC probe	37.9	24	5’-Thiol-GGTCC ACCAA ACGTA ATGCG GGGT-ATTO MB2-3’
SARS-CoV-2 N qPCR probe	37.9	24	GGTCC ACCAA ACGTA ATGCG GGGT
SARS-CoV-2 N EC target	46.1	72	GACCC CAAAA TCAGC GAAAT GCACC CCGCA TTACG TTTGG TGGAC CCTCA GATTC AACTG GCAGT AACCA GA
SARS-CoV-2 N qPCR target	63.7	24	ACCCC GCATT ACGTT TGGTG GACC
SARS-CoV-2 N F-primer	53.6	20	GACCC CAAAA TCAGC GAAAT
SARS-CoV-2 N R-primer	56.9	24	TCTGG TTACT GCCAG TTGAA TCTG
SARS-CoV-2 ORF 1ab EC probe	56.2	28	Thiol-CCATA ACCTT TCCAC ATACC GCAGA CGG-ATTO MB2
SARS-CoV-2 ORF 1ab qPCR probe	56.2	28	CCATA ACCTT TCCAC ATACC GCAGA CGG
SARS-CoV-2 ORF 1ab EC target	44.7	119	CCCTG TGGGT TTTAC ACTTA AAAAC ACAGT CTGTA CCGTC TGCGG TATGT GGAAA GGTTA TGGCT GTAGT TGTGA TCAAC TCCGC GAACC CATGC TTCAG TCAGC TGATG CACAA TCGT
SARS-CoV-2 ORF 1ab qPCR target	62.9	28	CCGTC TGCGG TATGT GGAAA GGTTA TGG
SARS-CoV-2 ORF 1ab F-primer	50.5	21	CCCTG TGGGT TTTAC ACTTA A
SARS-CoV-2 ORF 1ab R-primer	48.9	19	ACGAT TGTGC ATCAG CTGA
HCoV-229E GC MM	55.5	30	CATAG ATTGG CTTCA **T**GTGT TCAGG TTCAT
HCoV-229E 2 X GC MM	< 55.5	30	CATAG ATTGG CTTCA **TT**TGT TCAGG TTCAT
HCoV-229E AT MM	57.3	30	CATAG ATTGG CTTC**T** GGTGT TCAGG TTCAT

Table S2. Primers, targets, and probes for HCoV-229E and SARS-CoV-2 RNA detection. ATTO MB2 is a derivative of the well-known redox dye Methylene Blue**.** T_m refers to the temperature for dissociating double-strand DNA and opening the hairpin structure of a single-strand DNA probe or target (marked in blue color). Electrochemical (EC) target is the entire sequence that comprises the length of forward and reverse primers, and always longer than that of qPCR target equal to the length of its corresponding probe. The nucleotide marked in red color is the mismatch position of genomes.

Sample	Mode of detection / Company	Detection method (reference)	Response time	Sample-in-result-out time (approximate)	LoD	Assay result (quantitative)	Bulky device required
Clinical patient sample	Molecular technology (DNA or RNA gene)	RT-qPCR[1]	120 min	3.5 h (including RNA extraction)	3.2 copies/μl	Yes	Yes
		ddPCR[2]	120 min	3.5 h (including RNA extraction)	≤10 copies/μl	Yes	Yes
		RPA[3]	45 min	1.5 h (with manual RNA extraction)	100 - 300 copies / sample	- -	No
		RT-LAMP[4]	30 min	2 h (including RNA extraction)	0.44 copies/μl	- -	No
		CRISPR-Cas[5]	45 min	<1.5 h (with manual RNA extraction)	10 copies/μl	No	No
		FET[6]	2 min	1.5 h (including RNA extraction and PCR amplification)	223 copies/μl	- -	No
		LSPR[7]	30 min	<1 h (with manual RNA extraction)	166 copies/μl	- -	Yes
		Colorimetric assay[8]	30 min	1.5 h (with manual RNA extraction)	3.6 copies /sample	- -	No
		EC (this work)	10 min	<2 h (including RNA extraction and continuous-flow PCR amplification)	0.1 copy/μl	Yes	No
	Protein technology (S, N, lgM or lgG)	ELISA (Thermo Fisher Scientific) [9]	- -	2 h (including sample pretreatment)	1.953 ng/ml	No	Yes
		LFA[10]	15 min	<30 min (including sample pretreatment)	- -	- -	No
		MALDI-MS[11]	15 min	<30 min (including sample pretreatment)	- -	- -	Yes
		FET[6]	<2 min	<10 min (including sample pretreatment)	1 fg/ml	- -	No
		LSPR[12]	10 min	<20 min (including sample pretreatment)	4.2 fmol	- -	Yes
		SERS[13]	10 min	<20 min (including	10 PFU/ml	- -	Yes

				sample pretreatment)			
		EC[14]	<2 min	<10 min (including sample pretreatment)	≤1 ng/ml	- -	No
	Sequencing technology	Illumina	12 h	12 h (3072 COVID sample test)	1 ng/sample	Yes	Yes
		Nanopore[15]	6 – 10 h	6 – 10 h (including sample pretreatment)	10 copies / reaction	Yes	No
Airborne sample	Smith Group	Cellular Analysis	- -	3 min	6,000 PFU	- -	Yes
	ProGosis Biotech	ELISA	- -	2 h (including sample pretreatment)	0.55 ng/ml	No	Yes
	ACE Biolabs	LFA / PCR / RT-LAMP	- -	10 – 120 min	- -	- -	Yes
	Hayat Genetics Inc.	LAMP / Colorimetric	30 min	- -	- -	- -	Yes
	Sartorius	PCR	120 min	3.5 h (including RNA extraction)	- -	- -	Yes
	Bio-Rad	DdPCR[16]	120 min	3.5 h (including RNA extraction)	≤1 copy/sample	Yes	Yes
	ETH Zürich	EC (this work)	10 min	1.5 h (including RNA extraction and continuous-flow PCR amplification)	≤1 copy/m^3 (Method)	Yes	No

Table S3. Comparison of continuous-flow EC bioassay with other technologies for detecting SARS-CoV-2 virus from the patient and airborne samples. Abbreviations: FET, field-effect transistor; LSPR, localized surface plasmon resonance; SERS, surface-enhanced Raman spectroscopy; EC, Electrochemistry; MALDI-MS, matrix-assisted laser desorption/ionization mass spectrometry; Illumina, https://emea.illumina.com; Cellular Analysis, LAMP / Colorimetric, ELISA, https://www.acebiolab.com; LAMP, https://www.prognosis-biotech.com; Colorimetric, https://www.hayatgenetics.com; PCR, https://www.sartorius.com; PFU, plaque-forming unit.

HCoV-229 E virus (copies/µl)		0.3 m	0.6 m	0.9 m	1.2 m
Techniques	RT-qPCR	40	13	25	26
	EC bioassay	154	88	406	97

Table S4. Validation of analytical concordance for the quantitation of aerosolized HCoV-229E virus between RT-qPCR and EC bioassay. The results are in the same concentration class, demonstrating a close analytical concordance between the EC bioassay and RT-qPCR, indicating our prototype sensing platform capable of detecting airborne coronavirus accurately.

References

[1] C. B. F. Vogels, A. F. Brito, A. L. Wyllie, J. R. Fauver, I. M. Ott, C. C. Kalinich, M. E. Petrone, A. Casanovas-Massana, M. Catherine Muenker, A. J. Moore, J. Klein, P. Lu, A. Lu-Culligan, X. Jiang, D. J. Kim, E. Kudo, T. Mao, M. Moriyama, J. E. Oh, A. Park, J. Silva, E. Song, T. Takahashi, M. Taura, M. Tokuyama, A. Venkataraman, O. E. Weizman, P. Wong, Y. Yang, N. R. Cheemarla, E. B. White, S. Lapidus, R. Earnest, B. Geng, P. Vijayakumar, C. Odio, J. Fournier, S. Bermejo, S. Farhadian, C. S. Dela Cruz, A. Iwasaki, A. I. Ko, M. L. Landry, E. F. Foxman, N. D. Grubaugh, *Nat. Microbiol.* **2020**, *5* (10), 1299.

[2] H. N. Vasudevan, P. Xu, V. Servellita, S. Miller, L. Liu, A. Gopez, C. Y. Chiu, A. R. Abate, *Sci. Rep.* **2021**, *11* (1), 780.

[3] J. Qian, S. A. Boswell, C. Chidley, Z. X. Lu, M. E. Pettit, B. L. Gaudio, J. M. Fajnzylber, R. T. Ingram, R. H. Ward, J. Z. Li, M. Springer, *Nat. Commun.* **2020**, *11* (1), 5920.

[4] G. Xun, S. T. Lane, V. A. Petrov, B. E. Pepa, H. Zhao, *Nat. Commun.* **2021**, *12* (1), 2905.

[5] J. P. Broughton, X. Deng, G. Yu, C. L. Fasching, V. Servellita, J. Singh, X. Miao, J. A. Streithorst, A. Granados, A. Sotomayor-Gonzalez, K. Zorn, A. Gopez, E. Hsu, W. Gu, S. Miller, C. Y. Pan, H. Guevara, D. A. Wadford, J. S. Chen, C. Y. Chiu, *Nat. Biotechnol.* **2020**, *38* (7), 870.

[6] G. Seo, G. Lee, M. J. Kim, S. H. Baek, M. Choi, K. B. Ku, C. S. Lee, S. Jun, D. Park, H. G. Kim, S. J. Kim, J. O. Lee, B. T. Kim, E. C. Park, S. I. Kim, *ACS Nano* **2020**, *14* (4), 5135.

[7] G. Qiu, Z. Gai, L. Saleh, J. Tang, T. Gui, G. A. Kullak-Ublick, J. Wang, *ACS Nano* **2021**, *15* (4), 7536; b) N. Bhalla, A. F., Payam, A. Morelli, P. K., Sharma, R. Johnson, A. Thomson, P. Jolly, F. H., Canfarotta, *Sens. Actuators B Chem.* **2022**, *365*, 131906.

[8] P. Moitra, M. Alafeef, K. Dighe, M. B. Frieman, D. Pan, *ACS Nano* **2020**, *14* (6), 7617.

[9] R. Vernet, E. Charrier, J. Grogg, N. Mach, *Vaccines (Basel)* **2021**, *9* (7).

[10] L. J. Carter, L. V. Garner, J. W. Smoot, Y. Li, Q. Zhou, C. J. Saveson, J. M. Sasso, A. C. Gregg, D. J. Soares, T. R. Beskid, S. R. Jervey, C. Liu, *ACS Cent. Sci.* **2020**, *6* (5), 591.

[11] F. M. Nachtigall, A. Pereira, O. S. Trofymchuk, L. S. Santos, *Nat. Biotechnol.* **2020**, *38* (10), 1168.

[12] A. Ahmadivand, B. Gerislioglu, Z. Ramezani, A. Kaushik, P. Manickam, S. A. Ghoreishi, *Biosens. Bioelectron.* **2021**, *177*, 112971.

[13] H. Chen, S. G. Park, N. Choi, H. J. Kwon, T. Kang, M. K. Lee, J. Choo, *ACS Sens.* **2021**, *6* (6), 2378.

[14] R. M. Torrente-Rodriguez, H. Lukas, J. Tu, J. Min, Y. Yang, C. Xu, H. B. Rossiter, W. Gao, *Matter* **2020**, *3* (6), 1981.

[15] R. A. Bull, T. N. Adikari, J. M. Ferguson, J. M. Hammond, I. Stevanovski, A. G. Beukers, Z. Naing, M. Yeang, A. Verich, H. Gamaarachchi, K. W. Kim, F. Luciani, S. Stelzer-Braid, J. S. Eden, W. D. Rawlinson, S. J. van Hal, I. W. Deveson, *Nat. Commun.* **2020**, *11* (1), 6272.

[16] Y. Liu, Z. Ning, Y. Chen, M. Guo, Y. Liu, N. K. Gali, L. Sun, Y. Duan, J. Cai, D. Westerdahl, X. Liu, K. Xu, K. F. Ho, H. Kan, Q. Fu, K. Lan, *Nature* **2020**, *582* (7813), 557.
